# Effect of on-site first aid for industrial injuries on healthcare utilization after medical treatment: a 4-year retrospective longitudinal study

**DOI:** 10.1186/s12995-023-00380-8

**Published:** 2023-07-13

**Authors:** Jinhyun Kim, Hyunkyu Kim, Eun-Cheol Park, Sung-In Jang

**Affiliations:** 1 Department of Preventive Medicine & Institute of Health Services Research, Yonsei University College of Medicine, 50 Yonsei-ro, Seodaemun-gu, Seoul, 03722 Republic of Korea; 2 Institute of Health Services Research, Yonsei University, Seoul, Republic of Korea; 3 Department of Psychiatry, Yonsei University College of Medicine, Seoul, Republic of Korea

**Keywords:** Occupational safety, Outpatient, Hospitalization, Industrial injured workers, PSWCI

## Abstract

**Background:**

The number of industrially injured workers (IIW) is increasing in Korea. However, little research has been conducted on whether first aid is performed at industrial sites or on the association between first aid for industrial injuries and the prognosis of IIW, including healthcare utilization.

**Methods:**

A total of 3,092 participants (2,562 males and 530 females) were analyzed during the 4-year study period, which contributed to 11,167 observations. Healthcare utilization was evaluated based on the number of outpatient visits, hospitalizations, and duration of hospitalization using a generalized estimating equation Poisson regression. Several time-varying socioeconomic characteristics and information about the injury were adjusted, and transfer time to the medical institutions was also considered.

**Results:**

During 4-year after the termination of medical treatment, participants who had not receive first aid visited outpatient clinics 15.243 times per year, and those who had visited 13.928 times per year, which is 16.16% less (adjusted relative risk [aRR]: 0.838, 95% CI = 0.740–0.950). Participants who had received on-site first aid with less than a 0.5-hour transfer time to the medical institutions visited outpatient clinics 14.87% less per year than those who had not received first aid (aRR: 0.851, 95% CI = 0.750–0.966).

**Conclusion:**

To reduce the long-term outpatient utilization rate for IIW after medical treatment, on-site first aid must be provided in a timely manner. Employee education and first aid training are also necessary.

**Supplementary Information:**

The online version contains supplementary material available at 10.1186/s12995-023-00380-8.

## Background

According to the Occupational Safety and Health Act in Korea, industrial injuries is “any death, injury, or disease of a person who provides labor caused by structures, equipment, raw materials, gas, vapor, powder, dust, the other causes related to the duties, or by work or other duties” [1]. Based on the Ministry of Employment and Labor (MOEL), the incidence rate of industrial accidents in 2011 was 65 per 10,000 workers, and it remained almost the same in 2021 at 63 per 10,000 workers [2]. Meanwhile, the number of deaths due to industrial injuries was 2,114 in 2011 and 2,080 in 2021 [2]. The cumulative number of industrially injured workers (IIW) in South Korea has remained high; considering the non-reporting and concealment of accidents reported by Korea Occupational Safety and Health Agency (KOSHA), the actual figure could be even higher [3].

It is essential to minimize the consequences of industrial injuries from the perspectives of victims and the government. According to MOEL Korea, 23.5% of IIW received medical treatment for industrial injuries for more than 6 months and 60.8% of them were treated for 1–3 months in 2021 [4]. IIW suffer from chronic sequelae and diseases such as pneumoconiosis, hearing loss, brain damage, and musculoskeletal diseases [5]. As a result, the amount of insurance benefits (medical care, temporary layoff benefits, disability benefits, nursing benefits, survivors’ benefits, injury and disease compensation pensions, funeral expenses, and vocational rehabilitation) has increased from 3.62 trillion South Korean won (KRW) in 2011 to 6.45 trillion KRW in 2021 [6].

If industrial injuries occur, causing a layoff of three days or more, designated medical institutions provide medical treatment. After the conclusion of medical treatment, long-term care or medical rehabilitation for sequelae is provided, if medical necessity is proven. In the event of recurrence or aggravation of industrial injuries, the IIWs are eligible to receive additional medical treatment [7]. It is well known that on-site first aid provided to accident victims by bystanders or rescue workers is a substantial intervention for a good prognosis when there are delays in the arrival of medical professionals and transfers to the emergency room [8–11]. However, there has been little research on whether first aid for industrial injuries is performed at industrial sites. Further, the association between the provision of first aid for industrial injuries and the prognosis after medical treatment for IIW, including healthcare utilization, remains underexplored. Therefore, the primary objective of this study was to investigate the percentage of on-site first aid provision in the event of an industrial injury, focusing on industrial injuries and excluding occupational diseases and commuting accidents. In addition, this study analyzed throughout a 4-year period how on-site first aid is related to the rate of healthcare utilization (outpatient and hospitalization) after the provision of medical treatment for industrial injuries regardless of the severity of injuries. Furthermore, a detailed effect based on the transfer time from the accident site to the medical institutions was considered.

## Methods

### Study population and data

Panel Study of Workers’ Compensation Insurance (PSWCI) data from 2018 to 2021 by the Korea Workers’ Compensation & Welfare Service (KCOMWEL) were retrospectively analyzed. The PSWCI is a nationwide annual panel study of industrial injuries that collects data on industrial injuries insurance coverage and socioeconomic characteristics after the termination of medical treatment. The PSWCI is administered by tablet-assisted personal interviews (TAPI) with a visitor interview. In total, 81,252 IIW terminated medical treatment between January and December 2017. In the PSWCI, 3,294 participants among 81,252 IIW were included through proportional stratified random sampling based on disability grade (six categories), sex (two categories), age group (four categories: age below 30s, 40s, 50s, and above 60s), and systematic sampling based on area of residence (six categories) and rehabilitation service usage (two categories). In 2018, the experience of on-site first aid, disability level, and the duration of medical treatment for industrial injuries were evaluated. The number of outpatient visits and hospitalizations was assessed annually from 2018 to 2021. At the initial stage of analysis, 202 participants were excluded because they did not answer several questions regarding the variables. As a result, 3,092 participants (2,562 males and 530 females) were analyzed and contributed 11,167 observations during the 4-year study period. As of 2021, 2,797 participants remained in the PSWCI (retention rate: 84.9%).

### Measures

#### On-site first aid

The participants were asked whether they experienced on-site first aid by co-workers at the time of the industrial injuries and the response items were “yes” or “no.” According to the Occupational Safety and Health Act, business owners are obliged to prevent industrial injuries and provide employees with information on safety and health at relevant places of business, including tips for first aid [1]. Therefore, the KOSHA provides professional tips for first aid according to the type of industrial injuries [12]. For example, “Do not move the patient recklessly, and keep him/her in a comfortable position” is recommended as a general tip for fractured patients. In this study, industrial injuries involved accidents on duty, but not occupational diseases or commuting accidents.

#### Healthcare utilization: Outpatient and hospitalization

The participants were asked, “What is your history of visiting medical institutions in the past year?” The evaluation period for healthcare utilization extended from one year before the survey date to the survey date. Regardless of the reason for healthcare utilization, the number of outpatient visits, hospitalizations, and duration of hospitalization were evaluated. Pharmacies and nursing homes were excluded from the utilization count, and health checkups were excluded from the number of outpatient visits. Circuit outpatient visits were also excluded. For example, receiving treatment in more than two different departments at the same medical institution was only considered as one visit. If participants were admitted to the hospital for 365 days, and if they were admitted or discharged on the day of the emergency room visit, they were marked as hospitalized once. The total number of outpatient visits and hospitalization were counted because of study data limitations. According to Article 77 of the Industrial Accident Compensation Insurance Act in Korea, there is an additional medical care service provided for complications arising from occupational injuries [13]. However, only 43.14% of survey participants were aware of the existence of this service, as the beneficiaries are required to apply for it themselves. Furthermore, not all types and severities of complications are covered by this service (For example, the service exclusively covers facial nerve injuries that are classified as more severe than grade [12]. There is a specific list of 14 covered complications, and prior approval of medical necessity is required [14]. Therefore, to assess prognosis of industrial injuries after medical treatment, we considered the total outpatient visit and hospitalization count.

#### Transfer time to medical institutions

The participants were asked, “How long did it take for you to be transferred to the medical institution?” The transfer time to the medical institution was classified into four groups according to the PSWCI: less than 0.5 h, 0.5 ~ 1 h, 1–2 h, and more than 2 h.

### Covariates

Several time-varying socioeconomic and health-related characteristics were adjusted. Patients were classified as either male or female. The age group was divided according to the PSWCI: age below 30s, 40s, 50s, and above 60s. Education level was divided into university or higher, and high school graduation or lower. Current economic activity was divided into employed, unemployed, and economically inactive. Current household income was divided according to the quintile of household income. Household income is the sum of earned, financial, real estate, and other income. Statistics Korea data for the entire population were used as the income quintile cutoff for each year [15]. Area of residence was divided into metropolitan and provincial (rural). Medical histories of chronic diseases such as cancer, hypertension, and diabetes before industrial injuries were divided into without chronic disease and with chronic disease. The types of injuries due to industrial injuries were divided into fracture, sprain, back pain/musculoskeletal disease, amputation, cuts, bruising/concussion, rupture/laceration, burns, and other (abrasions, stab wounds, frostbite, contagion/addiction, and internal organ damage). Disability grade was based on 14 levels of physical/mental aftereffects of industrial injuries designated by law after the termination of primary medical care and symptom fixation [1]. The higher the disability grade, the more severe the disability level. In the analysis, disability grade was divided into the following groups as given by the PSWCI: grades 1–3 (most severe), 4–7, 8–9, 10–12, 13–14, and no disability. The period of primary medical care, which refers to the initial acute management at the designated hospital for IIW, was divided into the following as given by the PSWCI: < 3 months, 3–12 months, and > 1 year. Variables including sex, age, area of residence, type of injury, disability grade, and period of medical care were administrative data from the KCOMWEL, while variables including education level, current economic activity, current household income, and past medical history before accidents were self-reported.

### Statistical analysis

A generalized estimating equation (GEE) Poisson regression was applied for the analysis. Poisson regression with a log link function and an unstructured (UN) working correlation matrix, which had the lowest Quasi-likelihood under Independence Model Criterion (QIC) statistics, was used for the longitudinal data (from 2018 to 2021). The results are presented as adjusted relative risk (aRR) with 95% confidence intervals (CI). Subgroup analyses were conducted to determine the detailed effects based on transfer time to the medical institutions and the covariates. As the variance inflation factors (VIF) for all variables were less than 1.6, there was no evidence of multicollinearity. Version 9.4 SAS software (SAS Institute, Cary, North Carolina, USA) was used. Statistical significance was set at P ≤ 0.05.

## Results

The general characteristics of the participants in 2018 and the percentage of on-site first aid are presented in Table 1. Among 3,092 participants, a total of 1,252 participants (40.5%) did not receive on-site first aid, while 1,840 participants (59.5%) did. Among the participants who received on-site first aid, 2.6% were classified as disability grade 1–3 (indicating the most severe cases), and 14.5% required medical treatment for a duration exceeding one year. Among participants who did not receive first aid, the corresponding percentages were 1.6% and 11.4%, respectively. The most common type of injury in the total population was a fracture (60.8%). More than half of the participants had either grade 13–14 or no disability (51.8%), and had received medical care for 3–12 months (67.1%).

**Table 1:** General characteristics of study participants as of year 2018

Variables		No first aid		On-site first aid	
		N	(%)	N	(%)
**Total**		1252	(100.0)	1840	(100.0)
**Sex**					
	Male	1012	(80.8)	1550	(84.2)
	Female	240	(19.2)	290	(15.8)
**Age**					
	Below 30s	209	(16.7)	313	(17.0)
	40s	238	(19.0)	395	(21.5)
	50s	447	(35.7)	648	(35.2)
	Above 60s	358	(28.6)	484	(26.3)
**Education level**					
	High school graduation or lower	1007	(80.4)	1464	(79.6)
	University or higher	245	(19.6)	376	(20.4)
**Current economic activity**					
	Employed	380	(30.4)	559	(30.4)
	Unemployed	85	(6.8)	111	(6.0)
	Economically inactive population	787	(62.9)	1170	(63.6)
**Current household income**					
	Lowest quintile	127	(10.1)	146	(7.9)
	Second quintile	325	(26.0)	418	(22.7)
	Middle quintile	344	(27.5)	553	(30.1)
	Fourth quintile	301	(24.0)	465	(25.3)
	Top quintile	155	(12.4)	258	(14.0)
**Area of residence**					
	Metropolitan	618	(49.4)	877	(47.7)
	Province(rural)	634	(50.6)	963	(52.3)
**Past medical history before accident**					
	Without chronic disease	920	(73.5)	1414	(76.8)
	With chronic disease	332	(26.5)	426	(23.2)
**Type of injury**					
	Fracture	777	(62.1)	1102	(59.9)
	Sprained	27	(2.2)	20	(1.1)
	Back pain/Musculoskeletal disease	27	(2.2)	19	(1.0)
	Amputation	90	(7.2)	232	(12.6)
	Cuts	6	(0.4)	18	(1.0)
	Bruising/Concussion	34	(2.7)	19	(1.0)
	Rupture/Laceration	204	(16.3)	310	(16.8)
	Burns	31	(2.5)	66	(3.6)
	Others	56	(4.5)	54	(3.9)
**Disability grade**					
	Grade 1 ~ 3	20	(1.6)	48	(2.6)
	Grade 4 ~ 7	77	(6.2)	160	(8.7)
	Grade 8 ~ 9	125	(10.0)	218	(11.8)
	Grade 10 ~ 12	369	(29.5)	472	(25.7)
	Grade 13 ~ 14	383	(30.6)	583	(31.7)
	No disability	278	(22.2)	359	(19.5)
**Period of medicaltreatment**					
	Less than 3 months	269	(21.5)	337	(18.3)
	3 ~ 12 months	840	(67.1)	1236	(67.2)
	More than 1 year	143	(11.4)	267	(14.5)

The mean number of outpatient visits per year and the results of the GEE Poisson regression, which demonstrated the relationship between on-site first aid and healthcare utilization, are presented in Table 2. The “no first aid” and “on-site first aid” group had visited outpatient clinics 15.243 and 13.928 times per year (4-year average), respectively. Participants who had received on-site first aid visited outpatient clinics 16.16% less frequently per year (aRR: 0.838, 95% CI = 0.740–0.950) than those who had not. Compared to the year after medical treatment termination (2018), the number of outpatient visits was significantly lower in the second year or later (2019–2021). The lower the disability grade, the lower the number of outpatient visits, especially for participants with no disability, who visited outpatient clinics 76.04% less often than those with disability grades 1–3 (aRR: 0.240; 95% CI = 0.172–0.334). Participants who received more than 3 months of medical treatment visited outpatient clinics more frequently than those who received less than 3 months of medical care. There was no significant difference in the total number and duration of hospitalizations per year between the participants who did and did not receive first aid (**Supplementary Table **1).

**Table 2:** The relationship between on-site first aid and the number of outpatient visits

Variables		Mean (/year)	Total outpatient visits per year											
			Model 1				Model 2^a^				Model 3^b^			
			CrudeRR	95% CI			aRR	95% CI			aRR	95% CI		
**On-site first aid**														
	No first aid	15.243	1.000				1.000				1.000			
	On-site first aid	13.928	0.800*	0.699	-	0.916	0.840*	0.737	-	0.956	0.838*	0.740	-	0.950
**Year**														
	2018						1.000				1.000			
	2019						0.582*	0.503	-	0.673	0.577*	0.503	-	0.662
	2020						0.528*	0.455	-	0.613	0.527*	0.457	-	0.607
	2021						0.464*	0.404	-	0.533	0.465*	0.409	-	0.529
**Sex**														
	Male						1.000				1.000			
	Female						1.072	0.917	-	1.252	1.149	0.977	-	1.352
**Age**														
	Below 30s						1.000				1.000			
	40s						1.227	0.946	-	1.593	1.104	0.853	-	1.429
	50s						1.364*	1.106	-	1.683	1.213	0.983	-	1.498
	Above 60s						2.010*	1.590	-	2.542	1.709*	1.351	-	2.162
**Education level**														
	High school graduation or lower						1.000				1.000			
	University or higher						0.828*	0.708	-	0.969	0.847*	0.727	-	0.988
**Current economic activity**														
	Employed						1.000				1.000			
	Unemployed						0.952	0.751	-	1.207	0.947	0.757	-	1.184
	Economically inactive population						0.931	0.816	-	1.062	0.925	0.819	-	1.045
**Current household income**														
	Lowest quintile						1.000				1.000			
	Second quintile						0.844*	0.715	-	0.997	0.843*	0.717	-	0.991
	Middle quintile						0.770*	0.657	-	0.903	0.752*	0.645	-	0.876
	Fourth quintile						0.954	0.787	-	1.157	0.889	0.736	-	1.074
	Top quintile						0.911	0.727	-	1.142	0.815	0.657	-	1.011
**Area of residence**														
	Metropolitan						1.000				1.000			
	Province(rural)						1.192*	1.048	-	1.356	1.151*	1.014	-	1.306
**Past medical history before accident**														
	Without chronic disease						1.000				1.000			
	With chronic disease						1.272*	1.093	-	1.479	1.295*	1.116	-	1.502
**Type of injury**														
	Fracture										1.000			
	Sprained										1.181	0.884	-	1.577
	Back pain/Musculoskeletal disease										1.313	0.839	-	2.054
	Amputation										0.629	0.531	-	0.744
	Cuts										0.804	0.457	-	1.413
	Bruising/Concussion										1.191	0.709	-	2.001
	Rupture/Laceration										0.932	0.794	-	1.094
	Burns										0.793	0.510	-	1.232
	Others										0.860	0.676	-	1.094
**Disability grade**														
	Grade 1 ~ 3										1.000			
	Grade 4 ~ 7										0.400*	0.299	-	0.535
	Grade 8 ~ 9										0.379*	0.277	-	0.518
	Grade 10 ~ 12										0.336*	0.243	-	0.464
	Grade 13 ~ 14										0.236*	0.171	-	0.326
	No disability										0.240*	0.172	-	0.334
**Period of medical treatment**														
	Less than 3 months										1.000			
	3 ~ 12 months										1.285*	1.047	-	1.577
	More than 1 year										1.661*	1.281	-	2.154

Abbreviation: * Statistically significant; RR = Relative Risk; aRR = adjusted Relative Risk; CI = Confidence Interval

a Generalized estimating equation Poisson regression with adjustment for year, sex, age, education level, current economic activity, current household income, area of residence, and past medical history before accident

b Generalized estimating equation Poisson regression with adjustment for year, sex, age, education level, current economic activity, current household income, area of residence, past medical history before accident, type of injury, disability level, and period of medical treatment

The subgroup analyses based on covariates are presented in Table 3. The following subgroups showed significantly fewer outpatient visits per year for participants who received first aid compared to those who did not: in 2021 (4 years after medical treatment termination); males above 50 years of age; with a high school education or lower; currently employed; living in provinces; with a rupture/laceration and other types of injury; without disabilities; and had received less than 12 months of medical treatment.

**Table 3:** Subgroup analysis based on the general characteristics of participants: total number of outpatients visit

Variables		Total outpatients visit per year				
		No first aid	On-site first aid			
		aRR	aRR	95% CI		
**Year**						
	2018	1.000	0.900	0.777	-	1.043
	2019	1.000	0.827	0.682	-	1.002
	2020	1.000	0.844	0.663	-	1.073
	2021	1.000	0.717*	0.597	-	0.861
**Sex**						
	Male	1.000	0.822*	0.713	-	0.948
	Female	1.000	0.904	0.718	-	1.139
**Age**						
	Below 30s	1.000	1.103	0.818	-	1.488
	40s	1.000	0.977	0.721	-	1.326
	50s	1.000	0.768*	0.636	-	0.926
	Above 60s	1.000	0.799*	0.654	-	0.976
**Education level**						
	High school graduation or lower	1.000	0.825*	0.715	-	0.951
	University or higher	1.000	0.865	0.672	-	1.114
**Economic activity**						
	Employed	1.000	0.811*	0.685	-	0.960
	Unemployed	1.000	0.916	0.685		1.226
	Economically inactive population	1.000	0.891	0.762	-	1.040
**Economic status**						
	Lowest quintile	1.000	0.904	0.663	-	1.234
	s quintile	1.000	0.778*	0.642	-	0.942
	Middle quintile	1.000	0.897	0.778	-	1.035
	Fourth quintile	1.000	0.721*	0.571	-	0.909
	Top quintile	1.000	0.747	0.536		1.041
**Area of residence**						
	Metropolitan	1.000	0.936	0.797	-	1.099
	Province(rural)	1.000	0.791*	0.659	-	0.948
**Past medical history before accident**						
	Without chronic disease	1.000	0.868	0.740	-	1.017
	With chronic disease	1.000	0.842	0.696	-	1.018
**Type of injury**						
	Fracture	1.000	0.863	0.729	-	1.022
	Sprained	1.000	0.829	0.552	-	1.246
	Back pain/Musculoskeletal disease	1.000	0.396	0.138	-	1.137
	Amputation	1.000	0.947	0.718	-	1.250
	Cuts	1.000	0.779	0.301	-	2.013
	Bruising/Concussion	1.000	0.401	0.155	-	1.034
	Rupture/Laceration	1.000	0.749*	0.582	-	0.965
	Burns	1.000	1.133	0.747	-	1.718
	Others	1.000	0.903	0.638	-	1.279
**Disability grade**						
	Grade 1 ~ 3	1.000	N.A^a^			
	Grade 4 ~ 7	1.000	0.946	0.715	-	1.250
	Grade 8 ~ 9	1.000	1.129	0.830	-	1.536
	Grade 10 ~ 12	1.000	0.936	0.776	-	1.129
	Grade 13 ~ 14	1.000	0.849	0.702	-	1.027
	No disability	1.000	0.787*	0.643	-	0.962
**Period of medical treatment**						
	Less than 3 months	1.000	0.775*	0.614	-	0.979
	3 ~ 12 months	1.000	0.844*	0.719	-	0.990
	More than 1 year	1.000	1.311	0.992	-	1.731

a Due to insufficient number of samples

* Statistically significant; aRR = adjusted Relative Risk; CI = Confidence Interval; N.A = Not Applicable

**Fig. 1:**
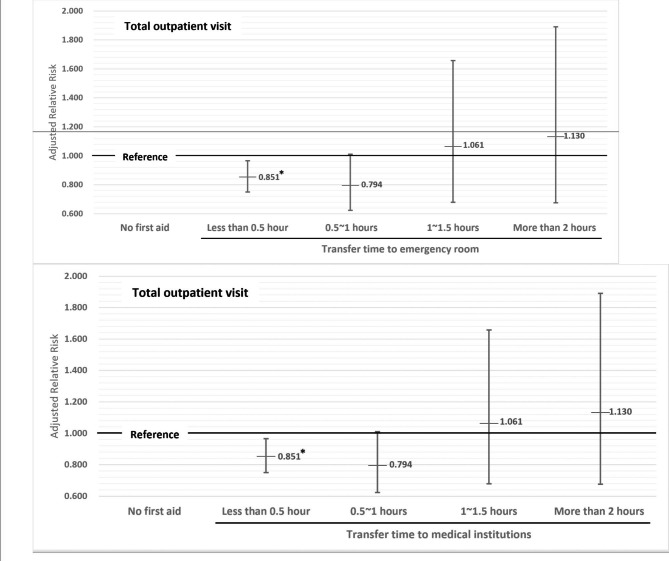
Subgroup analyses based on the transfer time to medical institutions: adjusted relative risk for total number of outpatients visits.^a^ * Statistically significant a Adjusted by year, sex, age, education level, economic activity, economic status, area of residence, past medical history before accident, type of injury, disability grade, period of medical treatment

The detailed effects of on-site first aid based on the transfer time to the medical institutions are presented in Fig. 1. Compared to participants who did not receive first aid, those who did with a transfer time of less than 0.5 h to the medical institutions visited outpatient clinics 14.87% less frequently per year (aRR: 0.851, 95% CI = 0.750–0.966).

## Discussion

This study examined the percentage of on-site first aid for industrial injuries among Korean workers from 2018 to 2021, and its effect on the rate of their healthcare utilization during a 4-year period after the termination of medical treatment. The study also considered the transfer time to the medical institutions.

After the termination of medical treatment for industrial injuries, IIW who did not receive on-site first aid visited outpatient clinics 15.243 times per year. Among the IIW, 59.5% received on-site first aid and, on average, visited outpatient clinics 13.928 times per year after medical treatment termination, which was 16.16% less than those who did not receive first aid (Table 2). When the transfer time to the medical institutions was more than 30 min, there was no significant difference in the outpatient visit rate between IIW who received first aid and those who did not (Fig. 1). Meanwhile, there were no significant differences in the total hospitalization count or duration (**Supplementary Table **1). Additionally, a decrease in the frequency of outpatient use was observed in subgroups of men over 50 years old living in provinces, indicating poorer medical surroundings compared to metropolitan areas and poorer health conditions (Table 3). Moreover, first aid significantly reduced the number of outpatient visits for industrial injuries that did not result in permanent disability and require less than one year of medical treatment, which was defined as relatively mild injuries in this study (Table 3). It is possible that first aid may not be effective in managing severe industrial accidents or that inappropriate first aid was provided to injured industrial workers. Therefore, further research is needed to investigate the effect of on-site first aid on the severely injured. On-site first aid for ruptures/lacerations was associated with fewer outpatient visits than that for other types of injuries (Table 3).

We adjusted the analyses for previously investigated factors associated with healthcare utilization, including income level, chronic diseases, advanced age, and being female [15–19]. Additionally, we adjusted for the type of injury and consequences of accidents. There has been a significant decrease in the number of outpatient visits after adjustment. This suggests that the on-site first aid administered may have played a role in preventing the exacerbation of the medical condition following acute-stage medical treatment.However, the association between the number of hospitalizations and on-site first aid suggests that on-site first aid, when not conducted by trained medical professionals, may not effectively reduce the risk of exacerbation of injuries to the extent that hospitalization is required. Receiving on-site first aid is crucial for reducing healthcare utilization after medical treatment; therefore, employees should receive first-aid training, especially for ruptures/lacerations. Having numerous workers who are capable of administering first aid can enhance preparedness for industrial injuries.

Furthermore, rapid access to medical facilities following injuries is crucial in a range of medical conditions [19–23]. Previous studies conducted in the United States have established that transferring patients to a hospital within 30 min is considered a criterion for expedited access to emergency services, and an average transfer duration of approximately 31 min observed in urban and suburban areas [24, 25]. In our research, we found that on-site first aid accompanied by shorter transfer times, specifically within 30 min, was associated with a reduced need for outpatient visits. While further investigation is warranted, our findings suggest that a 30-minute threshold for transfer time may be significant in the context of industrial injuries. Furthermore, having numerous workers who are capable of administering first aid can enhance preparedness for industrial injuries. This study had several limitations. First, some variables, including on-site first aid and healthcare utilization, were self-reported, which introduces the possibility of non-differential misclassification. This potential misclassification could lead to inaccurate results, including biases towards the null or away from the null hypothesis [26]. Moreover, owing to data unavailability, the study did not include detailed information on on-site first aid, such as the first-aid provider, severity of industrial injuries, and medical interventions during first aid. Although the participants’ past medical history was adjusted, the reasons for healthcare utilization were not considered due to the unavailability of data and insufficient coverage of national policies regarding the management of sequelae resulting from industrial injuries. Reverse causation is also a plausible explanation, as it is possible that participants who had a higher tendency to seek healthcare utilization could have requested on-site first aid following industrial injuries. Therefore, further research based on the precise reasons of healthcare utilization is needed.

Moreover, it should be noted that the on-site first aid was administered by coworkers, which raises concerns about the reliability and effectiveness of the provided first aid. Our results cannot be applied to other trauma events such as car accidents and cannot be generalized to other countries with different surroundings for industrial injuries.

## Conclusion

Despite its limitations, our study provides valuable insights into the potential benefits of on-site first aid in reducing healthcare utilization for industrial injuries. Specifically, the study found that first aid for ruptures/lacerations and relatively mild injuries, with less than 30 min of transfer time to the medical institutions, could reduce outpatient visits. Timely first-aid provision and employee education are necessary to reduce outpatient utilization rates.

## Electronic supplementary material

Below is the link to the electronic supplementary material.

**Supplementary Table 1** The relationship between on-site first aid and the number of hospitalization and duration of hospitalization

## Data Availability

The data analyzed in this study were obtained from the Panel Study of Workers’ Compensation Insurance (PSWCI), which is publicly available. All data were obtained from an official website (https://www.comwel.or.kr/Researchinstitute/index.do).

## List of abbreviations

IIW: Industrially injured workers
KRW: South Korean Won
PSWCI: Panel Study of Workers’ Compensation Insurance
KCOMWEL: Korea Workers’ Compensation & Welfare Service
TAPI: Tablet-assisted personal interviews
KOSHA: Korea Occupational Safety and Health Agency
GEE: Generalized estimating equation
QIC: Quasi-likelihood under Independence Model Criterion
aRR: Adjusted relative risk
CI: Confidence intervals
VIF: Variance inflation factors
