# Supplementary material for: Effect of on-site first aid for industrial injuries on healthcare utilization after medical treatment: a 4-year retrospective longitudinal study
Source: J Occup Med Toxicol. 2023 Jul 13;18:12. doi: 10.1186/s12995-023-00380-8 (PMC10339530; doi:10.1186/s12995-023-00380-8)
Supplement: Supplementary file 1 — Supplementary Table 1 The relationship between on-site first aid and the number of hospitalization and duration of hospitalization [file 12995_2023_380_MOESM1_ESM.docx]

**Supplementary Table 1** The relationship between on-site first aid and the number of hospitalization and duration of hospitalization.

| **Variables** | | **Mean**  **(/year)** | **Total number of hospitalizations per year** | | | | | | | | | | | | |
| --- | --- | --- | --- | --- | --- | --- | --- | --- | --- | --- | --- | --- | --- | --- | --- |
|  |  |  | **Model 1** | | | | **Model 2^a^** | | | | **Model 3^b^** | | | | |
|  |  |  | **Crude RR** | **95% CI** | | | **aRR** | **95% CI** | | | **aRR** | **95% CI** | | |  |
| **On-site first aid** | |  |  |  |  |  |  |  |  |  |  |  |  |  |  |
|  | No first aid | 0.208 | 1.000 |  |  |  | 1.000 |  |  |  | 1.000 |  |  |  |  |
|  | On-site first aid | 0.177 | 0.886 | 0.724 | - | 1.084 | 0.907 | 0.744 | - | 1.106 | 0.886 | 0.730 | - | 1.076 |  |
| **Year** | |  |  |  |  |  |  |  |  |  |  |  |  |  |  |
|  | 2018 |  |  |  |  |  | 1.000 |  |  |  | 1.000 |  |  |  |  |
|  | 2019 |  |  |  |  |  | 0.392* | 0.290 | - | 0.529 | 0.390* | 0.290 | - | 0.523 |  |
|  | 2020 |  |  |  |  |  | 0.520* | 0.382 | - | 0.707 | 0.518* | 0.383 | - | 0.700 |  |
|  | 2021 |  |  |  |  |  | 0.515* | 0.330 | - | 0.804 | 0.515* | 0.332 | - | 0.798 |  |
| **Sex** | |  |  |  |  |  |  |  |  |  |  |  |  |  |  |
|  | Male |  |  |  |  |  | 1.000 |  |  |  | 1.000 |  |  |  |  |
|  | Female |  |  |  |  |  | 0.887 | 0.711 | - | 1.108 | 0.915 | 0.729 | - | 1.148 |  |
| **Age** | |  |  |  |  |  |  |  |  |  |  |  |  |  |  |
|  | Below 30s |  |  |  |  |  | 1.000 |  |  |  | 1.000 |  |  |  |  |
|  | 40s |  |  |  |  |  | 0.957 | 0.720 | - | 1.271 | 0.931 | 0.698 | - | 1.242 |  |
|  | 50s |  |  |  |  |  | 1.261 | 0.937 | - | 1.696 | 1.200 | 0.883 | - | 1.632 |  |
|  | Above 60s |  |  |  |  |  | 1.116 | 0.847 | - | 1.471 | 1.040 | 0.782 | - | 1.383 |  |
| **Education level** | |  |  |  |  |  |  |  |  |  |  |  |  |  |  |
|  | High school graduation or lower |  |  |  |  |  | 1.000 |  |  |  | 1.000 |  |  |  |  |
|  | University or higher |  |  |  |  |  | 0.860 | 0.688 | - | 1.076 | 0.853 | 0.677 | - | 1.073 |  |
| **Current economic activity** | |  |  |  |  |  |  |  |  |  |  |  |  |  |  |
|  | Employed |  |  |  |  |  | 1.000 |  |  |  | 1.000 |  |  |  |  |
|  | Unemployed |  |  |  |  |  | 1.079 | 0.727 | - | 1.601 | 1.081 | 0.729 | - | 1.602 |  |
|  | Economically inactive population |  |  |  |  |  | 1.148 | 0.827 | - | 1.593 | 1.139 | 0.828 | - | 1.566 |  |
| **Current household income** | |  |  |  |  |  |  |  |  |  |  |  |  |  |  |
|  | Lowest quintile |  |  |  |  |  | 1.000 |  |  |  | 1.000 |  |  |  |  |
|  | Second quintile |  |  |  |  |  | 0.859 | 0.633 | - | 1.164 | 0.864 | 0.636 | - | 1.172 |  |
|  | Middle quintile |  |  |  |  |  | 0.756* | 0.587 | - | 0.974 | 0.754* | 0.583 | - | 0.975 |  |
|  | Fourth quintile |  |  |  |  |  | 0.745 | 0.537 | - | 1.034 | 0.727 | 0.526 | - | 1.005 |  |
|  | Top quintile |  |  |  |  |  | 0.528* | 0.360 | - | 0.774 | 0.507* | 0.345 | - | 0.746 |  |
| **Area of residence** | |  |  |  |  |  |  |  |  |  |  |  |  |  |  |
|  | Metropolitan |  |  |  |  |  | 1.000 |  |  |  | 1.000 |  |  |  |  |
|  | Province(rural) |  |  |  |  |  | 1.082 | 0.891 | - | 1.313 | 1.055 | 0.873 | - | 1.275 |  |
| **Past medical history before accident** | |  |  |  |  |  |  |  |  |  |  |  |  |  |  |
|  | Without chronic disease |  |  |  |  |  | 1.000 |  |  |  | 1.000 |  |  |  |  |
|  | With chronic disease |  |  |  |  |  | 1.306* | 1.056 | - | 1.615 | 1.313* | 1.063 | - | 1.622 |  |
| **Type of injury** | |  |  |  |  |  |  |  |  |  |  |  |  |  |  |
|  | Fracture |  |  |  |  |  |  |  |  |  | 1.000 |  |  |  |  |
|  | Sprained |  |  |  |  |  |  |  |  |  | 0.734 | 0.416 | - | 1.296 |  |
|  | Back pain/Musculoskeletal disease |  |  |  |  |  |  |  |  |  | 0.765 | 0.391 | - | 1.496 |  |
|  | Amputation |  |  |  |  |  |  |  |  |  | 0.773 | 0.592 | - | 1.008 |  |
|  | Cuts |  |  |  |  |  |  |  |  |  | 0.861 | 0.388 | - | 1.912 |  |
|  | Bruising/Concussion |  |  |  |  |  |  |  |  |  | 0.819 | 0.484 | - | 1.388 |  |
|  | Rupture/Laceration |  |  |  |  |  |  |  |  |  | 1.088 | 0.778 | - | 1.521 |  |
|  | Burns |  |  |  |  |  |  |  |  |  | 0.741 | 0.409 | - | 1.341 |  |
|  | Others |  |  |  |  |  |  |  |  |  | 1.059 | 0.632 | - | 1.775 |  |
| **Disability grade** | |  |  |  |  |  |  |  |  |  |  |  |  |  |  |
|  | Grade 1~3 |  |  |  |  |  |  |  |  |  | 1.000 |  |  |  |  |
|  | Grade 4~7 |  |  |  |  |  |  |  |  |  | 0.335* | 0.152 | - | 0.737 |  |
|  | Grade 8~9 |  |  |  |  |  |  |  |  |  | 0.405* | 0.184 | - | 0.892 |  |
|  | Grade 10~12 |  |  |  |  |  |  |  |  |  | 0.322* | 0.146 | - | 0.712 |  |
|  | Grade 13~14 |  |  |  |  |  |  |  |  |  | 0.294* | 0.132 | - | 0.658 |  |
|  | No disability |  |  |  |  |  |  |  |  |  | 0.348* | 0.151 | - | 0.799 |  |
| **Period of primary medical care** | |  |  |  |  |  |  |  |  |  |  |  |  |  |  |
|  | Less than 3 months |  |  |  |  |  |  |  |  |  | 1.000 |  |  |  |  |
|  | 3~12 months |  |  |  |  |  |  |  |  |  | 1.149 | 0.857 | - | 1.542 |  |
|  | More than 1 year |  |  |  |  |  |  |  |  |  | 1.296 | 0.856 | - | 1.962 |  |
| **Variables** | | **Mean (days/year)** | **Total duration of hospitalization per year** | | | | | | | | | | | | |
|  |  |  | **Model 1** | | | | **Model 2** | | | | **Model 3** | | | | |
|  |  |  | **Crude RR** | **95% CI** | | | **aRR** | **95% CI** | | | **aRR** | **95% CI** | | |  |
| **On-site first aid** | |  |  |  |  |  |  |  |  |  |  |  |  |  |  |
|  | No first aid | 4.745 | 1.000 |  |  |  | 1.000 |  |  |  | 1.000 |  |  |  |  |
|  | On-site first aid | 3.751 | 0.901 | 0.714 | - | 1.137 | 0.909 | 0.721 | - | 1.145 | 0.894 | 0.712 | - | 1.124 |  |
| **Year** | |  |  |  |  |  |  |  |  |  |  |  |  |  |  |
|  | 2018 |  |  |  |  |  | 1.000 |  |  |  | 1.000 |  |  |  |  |
|  | 2019 |  |  |  |  |  | 0.285* | 0.194 | - | 0.419 | 0.282* | 0.196 | - | 0.405 |  |
|  | 2020 |  |  |  |  |  | 0.315* | 0.224 | - | 0.443 | 0.310* | 0.226 | - | 0.427 |  |
|  | 2021 |  |  |  |  |  | 0.306* | 0.206 | - | 0.454 | 0.303* | 0.207 | - | 0.443 |  |
| **Sex** | |  |  |  |  |  |  |  |  |  |  |  |  |  |  |
|  | Male |  |  |  |  |  | 1.000 |  |  |  | 1.000 |  |  |  |  |
|  | Female |  |  |  |  |  | 0.660* | 0.502 | - | 0.869 | 0.707* | 0.528 | - | 0.947 |  |
| **Age** | |  |  |  |  |  |  |  |  |  |  |  |  |  |  |
|  | Below 30s |  |  |  |  |  | 1.000 |  |  |  | 1.000 |  |  |  |  |
|  | 40s |  |  |  |  |  | 0.940 | 0.631 | - | 1.402 | 0.872 | 0.585 | - | 1.301 |  |
|  | 50s |  |  |  |  |  | 1.081 | 0.746 | - | 1.568 | 0.954 | 0.653 | - | 1.393 |  |
|  | Above 60s |  |  |  |  |  | 1.333 | 0.904 | - | 1.965 | 1.117 | 0.741 | - | 1.684 |  |
| **Education level** | |  |  |  |  |  |  |  |  |  |  |  |  |  |  |
|  | High school graduation or lower |  |  |  |  |  | 1.000 |  |  |  | 1.000 |  |  |  |  |
|  | University or higher |  |  |  |  |  | 0.702* | 0.508 | - | 0.970 | 0.725* | 0.529 | - | 0.993 |  |
| **Current economic activity** | |  |  |  |  |  |  |  |  |  |  |  |  |  |  |
|  | Employed |  |  |  |  |  | 1.000 |  |  |  | 1.000 |  |  |  |  |
|  | Unemployed |  |  |  |  |  | 1.145 | 0.597 | - | 2.198 | 1.146 | 0.597 | - | 2.200 |  |
|  | Economically inactive population |  |  |  |  |  | 1.230 | 0.885 | - | 1.709 | 1.244 | 0.918 | - | 1.685 |  |
| **Current household income** | |  |  |  |  |  |  |  |  |  |  |  |  |  |  |
|  | Lowest quintile |  |  |  |  |  | 1.000 |  |  |  | 1.000 |  |  |  |  |
|  | Second quintile |  |  |  |  |  | 1.048 | 0.790 | - | 1.392 | 1.040 | 0.781 | - | 1.387 |  |
|  | Middle quintile |  |  |  |  |  | 0.862 | 0.638 | - | 1.165 | 0.829 | 0.605 | - | 1.135 |  |
|  | Fourth quintile |  |  |  |  |  | 0.841 | 0.557 | - | 1.269 | 0.753 | 0.495 | - | 1.146 |  |
|  | Top quintile |  |  |  |  |  | 1.102 | 0.717 | - | 1.695 | 0.904 | 0.590 | - | 1.386 |  |
| **Area of residence** | |  |  |  |  |  |  |  |  |  |  |  |  |  |  |
|  | Metropolitan |  |  |  |  |  | 1.000 |  |  |  | 1.000 |  |  |  |  |
|  | Province(rural) |  |  |  |  |  | 1.243 | 0.991 | - | 1.559 | 1.193 | 0.957 | - | 1.486 |  |
| **Past medical history before accident** | |  |  |  |  |  |  |  |  |  |  |  |  |  |  |
|  | Without chronic disease |  |  |  |  |  | 1.000 |  |  |  | 1.000 |  |  |  |  |
|  | With chronic disease |  |  |  |  |  | 1.195 | 0.919 | - | 1.554 | 1.216 | 0.937 | - | 1.579 |  |
| **Type of injury** | |  |  |  |  |  |  |  |  |  |  |  |  |  |  |
|  | Fracture |  |  |  |  |  |  |  |  |  | 1.000 |  |  |  |  |
|  | Sprained |  |  |  |  |  |  |  |  |  | 0.663 | 0.254 | - | 1.733 |  |
|  | Back pain/Musculoskeletal disease |  |  |  |  |  |  |  |  |  | 0.720 | 0.287 | - | 1.808 |  |
|  | Amputation |  |  |  |  |  |  |  |  |  | 0.737 | 0.461 | - | 1.178 |  |
|  | Cuts |  |  |  |  |  |  |  |  |  | 0.562 | 0.264 | - | 1.198 |  |
|  | Bruising/Concussion |  |  |  |  |  |  |  |  |  | 0.439* | 0.204 | - | 0.941 |  |
|  | Rupture/Laceration |  |  |  |  |  |  |  |  |  | 0.588* | 0.430 | - | 0.804 |  |
|  | Burns |  |  |  |  |  |  |  |  |  | 0.729 | 0.358 | - | 1.487 |  |
|  | Others |  |  |  |  |  |  |  |  |  | 1.095 | 0.538 | - | 2.231 |  |
| **Disability grade** | |  |  |  |  |  |  |  |  |  |  |  |  |  |  |
|  | Grade 1~3 |  |  |  |  |  |  |  |  |  | 1.000 |  |  |  |  |
|  | Grade 4~7 |  |  |  |  |  |  |  |  |  | 0.305* | 0.147 | - | 0.633 |  |
|  | Grade 8~9 |  |  |  |  |  |  |  |  |  | 0.364* | 0.169 | - | 0.783 |  |
|  | Grade 10~12 |  |  |  |  |  |  |  |  |  | 0.350* | 0.156 | - | 0.781 |  |
|  | Grade 13~14 |  |  |  |  |  |  |  |  |  | 0.255* | 0.112 | - | 0.584 |  |
|  | No disability |  |  |  |  |  |  |  |  |  | 0.265* | 0.116 | - | 0.602 |  |
| **Period of primary medical care** | |  |  |  |  |  |  |  |  |  |  |  |  |  |  |
|  | Less than 3 months |  |  |  |  |  |  |  |  |  | 1.000 |  |  |  |  |
|  | 3~12 months |  |  |  |  |  |  |  |  |  | 1.215 | 0.850 | - | 1.737 |  |
|  | More than 1 year |  |  |  |  |  |  |  |  |  | 1.613 | 0.902 | - | 2.884 |  |

Abbreviation: * Statistically significant; RR=Relative Risk; aRR=adjusted Relative Risk; CI=Confidence Interval.

a Generalized estimating equation Poisson regression with adjustment for year, sex, age, education level, current economic activity, current household income, area of residence, and past medical history before accident.

b Generalized estimating equation Poisson regression with adjustment for year, sex, age, education level, current economic activity, current household income, area of residence, past medical history before accident, type of injury, disability level, and period of medical treatment.
